# A call to improve understanding of Undetectable equals Untransmittable (U = U) in Brazil: a web‐based survey

**DOI:** 10.1002/jia2.25630

**Published:** 2020-11-06

**Authors:** Thiago S Torres, Joseph Cox, Luana MS Marins, Daniel RB Bezerra, Valdilea G Veloso, Beatriz Grinsztejn, Paula M Luz

**Affiliations:** ^1^ Instituto Nacional de Infectologia Evandro Chagas Fundação Oswaldo Cruz Rio de Janeiro Brazil; ^2^ Department of Epidemiology, Biostatistics and Occupational Health Faculty of Medicine McGill University Montreal Canada; ^3^ Research Institute McGill University Health Centre Montreal Canada

**Keywords:** HIV, key and vulnerable populations, men who have sex with men, Brazil, U = U slogan, treatment as prevention

## Abstract

**Introduction:**

Currently, the slogan “Undetectable = Untransmittable” (U = U), launched to disseminate scientific evidence on how people living with HIV (PLHIV) on antiretroviral treatment with suppressed viral load cannot transmit HIV to their sexual partners, is still challenged by individuals with differential acceptance across populations. In this study, we documented the perceived accuracy of U = U in Brazil in three different groups: PLHIV, HIV‐negative/unknown cisgender gay/bisexual men who have sex with men (GBM) and HIV‐negative/unknown other populations (POP).

**Methods:**

Adult (age ≥ 18y) Brazilians were recruited during October 2019 to complete a web‐based survey advertised on Grindr, Facebook and WhatsApp. Perceived accuracy of U = U was assessed with the question: “With regards to HIV‐positive individuals transmitting HIV through sexual contact, how accurate do you believe the slogan U = U is?” Response options ranged from 1 (Completely inaccurate) to 4 (Completely accurate) plus a fifth option (I don’t know what “undetectable” means). Participants’ characteristics were described according to perceived accuracy of U = U. Logistic regression models assessed the factors associated with perceived accuracy of U = U (completely accurate vs. partially accurate/inaccurate or completely inaccurate) by group.

**Results:**

Of 2311 individuals accessing the questionnaire, 1690 (73.1%) met inclusion/exclusion criteria and completed it. Of these, 347 (20.5%) were PLHIV, 785 (46.4%) GBM and 558 (33.0%) POP. More PLHIV perceived U = U as completely accurate (79.0%), compared to 44.2% GBM and 17.2% POP (*p* < 0.001). Among PLHIV, Black identity was associated with decreased odds of perceiving U = U as completely accurate while having a steady partner was associated with increased odds. Among GBM, being gay, having middle/higher income, being a resident of state capital metropolitan areas and ever testing for HIV were associated with increased odds. Lastly, among POP, ever testing for HIV increased the odds of perceiving U = U as completely accurate.

**Conclusions:**

There was a significant difference in perceived accuracy of U = U across population groups. Accurate understanding of the slogan needs to be promoted in more vulnerable populations such as PLHIV of Black identity and GBM of lower income to maximize individual and societal prevention benefits. Moreover, broader understating of U = U among the general population can help decrease societal stigma towards PLHIV.

## 1 Introduction

Over the past decade, an increasing body of knowledge has demonstrated the effectiveness of treatment as prevention (TasP), wherein the use of antiretroviral treatment among people living with HIV (PLHIV) reduces HIV transmission yielding public health as well as personal health benefits. Evidence has accumulated for both heterosexual and same‐sex couples using a variety of study designs, from ecological studies of population viral load leading to reduced transmission [1], to observational studies [2, 3], and randomized clinical trials [4] showing no linked HIV transmissions. In 2016, the slogan “Undetectable = Untransmittable” (U = U) was launched by the Prevention Access Campaign to translate scientific evidence into a community message that highlights how PLHIV on antiretroviral treatment with suppressed viral load cannot transmit HIV to their sexual partners [5]. Several publications summarized the scientific consensus providing guidelines for clinical practice and for use of the U = U message [6, 7].

In the interim, studies have shown that the slogan U = U, has not been clearly communicated by health providers in clinical care [8]. A qualitative study conducted in Kenya of HIV serodiscordant couples attending public clinics, showed how healthcare providers lacked “in‐depth knowledge and conviction” on U = U [9]. Similarly, studies conducted in high‐income settings (Canada [10, 11], Italy [12], Australia [13] and the United States [14, 15, 16]), mostly on gay, bisexual and other men who have sex with men, have reported low awareness, understanding, belief or perceived accuracy of TasP or U = U, depending on the study. In the largest study thus far conducted among sexual minority men from the United States, perceived accuracy of U = U was shown to vary by HIV status: 84% of HIV‐positive men rated U = U as accurate compared to 54% and 39% of HIV‐negative and status unknown men, respectively [14].

Thus far, published studies have primarily documented perceived accuracy of U = U among gay, bisexual and other men who have sex with men with only one study also including heterosexual men and women reporting condomless sexual intercourse in the last 12 months with a casual partner [12]. Research focused on populations more vulnerable to HIV is highly significant as U = U empowers PLHIV while reducing fear, guilt and HIV‐related self‐stigma [6]. However, understanding of U = U more broadly could help reduce societal stigma towards PLHIV [17, 18, 19] and thus increase population health given stigma’s role as a fundamental cause of health inequalities [20, 21].

In Brazil, the Ministry of Health officially endorsed the slogan U = U (highlighting that PLHIV with an undetectable viral load do not transmit HIV to sexual partners) on 15 May 2019 with formal guidance for healthcare providers to educate the public on the scientific evidence [22]. Since then, non‐governmental, civil society and region‐wide research collaborations such as ImPrEP (PrEP Implementation Project in Brazil, Peru and Mexico) have advertised U = U using social media and dating apps, including Facebook and Grindr, mostly targeting key populations, such as gay, bisexual and other men who have sex with men. However, as evidence from other settings suggest, it may well be that the U = U prevention message is not being heard and that its comprehension is highly differential. Therefore, we aimed to document perceived accuracy of U = U in Brazil in populations similar to those of previous studies in addition to others not previously assessed.

## 2 Methods

From 01 to 31 October 2019, an anonymous web‐based survey in Brazilian Portuguese was advertised using a geosocial networking (GSN) dating app (Grindr), Facebook social media and WhatsApp. We used banners targeting cisgender gay, bisexual or other men who have sex with men for advertisement on Grindr. On Facebook, we created a post on the Instituto Nacional de Infectologia Evandro Chagas (INI‐FIOCRUZ) fan page with the link to the survey; no specific population was targeted. Members of INI‐FIOCRUZ’s Community Advisory Board shared the survey link on WhatsApp Groups, targeting mostly cis and trans women living with HIV; respondents were encouraged to share the survey link among partners and friends. Individuals who met the eligibility criteria (Brazilian residents aged ≥18 years) and who acknowledged reading the informed consent text were directed to the questionnaire. Exclusion criteria were: self‐report of completing the questionnaire previously and an incorrect answer to any one of the three attention questions that were included throughout the questionnaire after 15 to 20 items; the assumption is that participants who answered these items erroneously were not paying attention [23]. This was a convenience sample with respondents from all five administrative regions of Brazil; the objective was to reach the maximum number of participants as possible without a specific sample size calculation. This study was approved by INI‐FIOCRUZ institutional review board (#CAAE 01777918.0.0000.5262) in accordance with all applicable regulations. No incentives were provided for completing the survey and, on average, participants took five minutes to do so.

The survey instrument was composed of 17 questions addressing: sociodemographics, gender, sexual orientation and HIV‐testing/status. Additionally, perceived accuracy of the prevention benefits of U = U was assessed with the question: “With regards to HIV‐positive individuals transmitting HIV through sexual contact, how accurate do you believe the slogan U = U is?” as used in previous studies [14, 24], though we added an explanation in our translated version stating “meaning that people who have HIV but have undetectable viral load do not transmit HIV through sex.” Response options were based on a Likert‐type scale from 1 (Completely inaccurate) to 4 (Completely accurate) plus a fifth option (I don’t know what “undetectable” means).

Participants’ answers to the gender, sexual orientation and HIV status items allowed us to group the study population into three mutually exclusive groups: people living with HIV (PLHIV), HIV‐negative/unknown cisgender gay or bisexual men who have sex with men (GBM) and HIV‐negative/unknown other populations (POP). The rationale for this *a priori* categorization was: 1) prior studies show that perceived accuracy of U = U differs by HIV status (PLHIV show greater perceived accuracy), 2) our interest in populations not addressed in prior studies (cisgender women and heterosexual men not living with HIV) and 3) that predictors of perceived accuracy would differ between the groups. Other variables included age at the time of the survey, race, monthly family income, education, residence in state capital metropolitan area, steady partner and ever tested for HIV.

Participants’ characteristics were described overall and according to perceived accuracy of U = U for each study population group. We used chi‐square tests to compare, across groups, the proportions of participants endorsing each of the five response options of the perceived accuracy of U = U item. Logistic regression models were used to assess the factors associated with perceived accuracy of U = U (completely accurate vs. partially accurate/inaccurate or completely inaccurate) by group. Given the ordinal nature of the response scale, a second analysis using ordinal logistic regression models was done to explore the factors associated with perceived accuracy ratings by group, similar to previous studies on the topic [14, 15]. In both regression analyses, those reporting not knowing what undetectable meant were removed. Analyses were performed using R (The R project www.r‐project.org, version 4.0.1).

## 3 Results and Discussion

A total of 2311 individuals accessed the questionnaire, 28 (1.2%) did not meet inclusion criteria, 206 (8.9%) met exclusion criteria and from the 2077 eligible, 387 (16.7%) did not complete the survey. Out of the 1690 (73.1%) who completed the survey, 347 (20.5%) were PLHIV, 785 (46.4%) GBM and 558 (33.0%) POP. Most participants were from the Southeast of Brazil (990/1690, 58.6%), where São Paulo and Rio de Janeiro are located, followed by Northeast (271/1690, 16.0%), South (255/1690, 15.1%), Central‐West (112/1690, 6.6%) and North (62/1690, 3.7%). Half of the participants were recruited on Grindr (850/1690; 50.3%), followed by Facebook (655/1690, 38.8%) and WhatsApp (185/1690, 10.9%).

GBM were younger (median age 33 years [interquartile range (IQR): 26 to 41]) compared to PLHIV (median 40 years, IQR: 32 to 49) and POP (median 48 years, IQR: 35 to 61). Overall, most of participants identified as White (961/1662, 57.8%), followed by *Pardo* or mixed (518/1662, 31.2%) and Black (183/1662, 11.0%). The majority had middle/high income (1149/1690, 68.0%), education greater than secondary level (1097/1681, 65.3%) and were residents of state capitals’ metropolitan areas (1194/1690, 70.7%). Most POP had a steady partner (357/558, 64.0%) while a much smaller fraction was observed for PLHIV (127/327, 36.6%) and GBM (223/785, 28.4%). More GBM reported ever testing for HIV (664/785, 84.6%) than POP (387/558, 69.4%).

**Figure 1:**
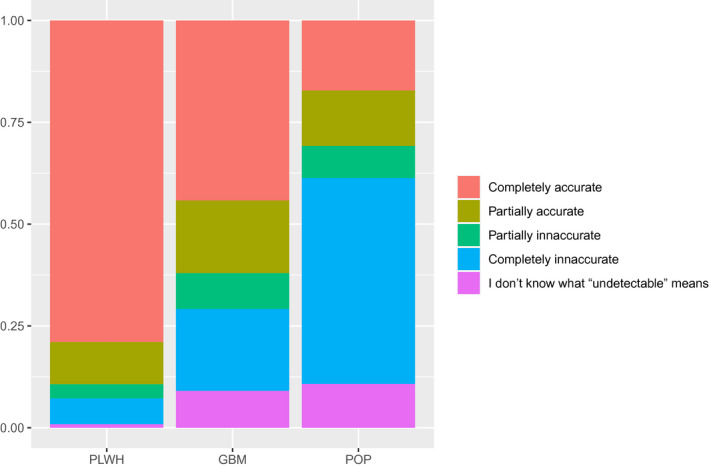
Proportion of participants who rated the item “With regards to HIV‐positive individuals transmitting HIV through sexual contact, how accurate do you believe the slogan U = U is?” as Completely inaccurate to Completely accurate, or who said they did not know what “undetectable” meant by population group: people living with HIV (PLHIV), HIV‐negative/unknown cisgender gay or bisexual men who have sex with men (GBM) and HIV‐negative/unknown other populations (POP).

As shown in Figure 1, more PLHIV perceived U = U as completely accurate (79.0%, 274/347), compared to 44.2% (347/785) GBM and 17.2% (96/558) POP (chi‐square test *p* < 0.001). Similarly, the fraction of individuals reporting “not knowing what undetectable is” varied by group with only three (0.9%, 3/347) reporting it among PLHIV compared to quite similar proportions among GBM (9.0%, 71/785) and POP (10.8%, 60/558) (chi‐square test *p* < 0.001).

Results from the logistic regression models indicated that among PLHIV, Black identity was associated with decreased odds of perceiving U = U as completely accurate while having a steady partner was associated with increased odds (Table 1). Among GBM, being gay, having middle/higher income, being a resident of state capital metropolitan areas and ever testing for HIV were associated with increased odds of perceiving U = U as completely accurate. Lastly, among POP, ever testing for HIV increased the odds of perceiving U = U as completely accurate. In the secondary analysis, the direction and magnitude of association of factors with perceived accuracy of U = U were overall the same, but younger age increased the odds of perceived accuracy of U = U across all groups, including POP (Table 2).

**Table 1:** Factors associated with perceived accuracy of the U = U slogan among mutually exclusive population groups defined by participant’s answers to the gender, sexual orientation and HIV status items: people living with HIV (PLHIV), HIV‐negative/unknown gay/homosexual, bisexual or other cisgender men who have sex with men (GBM) and HIV‐negative/unknown other populations (POP), Brazil, 2019

	Overall	PLHIV (N = 347/1690, 20.5%)		GBM (N = 785/1690, 46.5%)		POP (N = 558/1690, 33.0%)	
	N = 1690 (%)	Rated completely accurate 274 (79.0%)	aOR (95%CI)	Rated completely accurate 347 (44.2%)	aOR (95%CI)	Rated completely accurate 96 (17.2%)	aOR (95%CI)
Gender							
Cisgender men	1159 (68.6)	214 (79.3)	Ref.	NA	NA	16 (15.4)	Ref.
Cisgender women	497 (29.4)	51 (78.5)	0.83 (0.19 to 3.31)	NA	NA	71 (16.4)	1.02 (0.55 to 1.95)
Transgender/ non‐binary	34 (2.0)	9 (75.0)	0.84 (0.18 to 6.16)	NA	NA	9 (40.9)	3.10 (0.96 to 10.14)
Sexual orientation							
Gay	885 (52.6)	200 (79.4)	1.69 (0.55 to 4.64)	295 (48.5)	**2.40 (1.63 to 3.56)** ****	3 (12.0)	0.25 (0.05 to 0.98)
Bisexual	241 (14.3)	16 (69.6)	Ref.	52 (29.9)	Ref.	14 (31.8)	Ref.
Heterosexual	557 (33.1)	56 (81.2)	4.18 (0.77 to 24.26)	0		78 (16.0)	0.53 (0.24 to 1.18)
Age							
≤35 years	730 (43.2)	109 (86.5)	**3.23 (1.68 to 6.56)** ****	229 (49.6)	**1.88 (1.36 to 2.61)** ****	30 (21.1)	1.09 (0.62 to 1.87)
>35 years	960 (56.8)	165 (74.7)	Ref.	118 (36.5)	Ref.	66 (15.9)	Ref.
Ethno‐racial identity							
Black	183 (11.0)	30 (63.8)	**0.28 (0.13 to 0.61)** ****	43 (47.3)	1.42 (0.87 to 2.33)	8 (17.8)	0.78 (0.31 to 1.76))
Other^a^	1479 (89.0)	239 (81.0)	Ref.	300 (43.8)	Ref.	87 (17.8)	Ref.
Monthly family income							
Middle/High	1149 (68.0)	173 (82.4)	1.59 (0.83 to 3.05)	250 (47.4)	**1.51 (1.06 to 2.18)** **	65 (15.8)	0.73 (0.42 to 1.29)
Low^b^	541 (32.0)	101 (73.7)	Ref.	97 (37.6)	Ref.	31 (21.2)	Ref.
Education							
≤Secondary level	584 (34.7)	112 (77.2)	0.98 (0.51 to 1.89)	100 (41.0)	1.13 (0.78 to 1.64)	36 (18.5)	1.09 (0.63 to 1.86)
>Secondary level	1097 (65.3)	161 (80.5)	Ref.	246 (45.9)	Ref.	60 (16.6)	Ref.
Residence in state capital metro area							
Yes	1194 (70.7)	205 (79.8)	1.48 (0.77 to 2.80)	269 (46.6)	**1.60 (1.13 to 2.28)** ***	67 (13.4)	1.15 (0.70 to 1.93)
No	496 (29.3)	69 (76.7)	Ref.	78 (37.5)	Ref.	29 (14.6)	Ref.
Steady partner							
Yes	707 (41.8)	111 (87.4)	**2.84 (1.48 to 5.80)** ***	103 (46.2)	1.16 (0.83 to 1.64)	69 (19.3)	1.30 (0.77 to 2.23)
No	983 (58.2)	163 (74.1)	Ref.	244 (43.4)	Ref.	27 (13.4)	Ref.
Ever tested for HIV							
Yes	1338 (82.7)	NA	NA	305 (45.9)	1.29 (0.81 to 2.05)	80 (20.7)	**2.21 (1.23 to 4.17)** **
No	292 (17.3)	NA	NA	42 (34.7)	Ref.	16 (9.4)	Ref.

NA, not applicable.

Bold indicates statistical significance (*p* < .05).

^a^ Other = White, Asian, Native or Pardo (Mixed‐Black) identities

^b^ low income is equivalent to R$1996.00 or USD468.00 per month.

**** *p* < .0001

*** *p* < .001

** *p* < .01

**Table 2:** Factors associated with increasing perceived accuracy of the U = U slogan among mutually exclusive population groups defined by participant’s answers to the gender, sexual orientation and HIV status items: people living with HIV (PLHIV), HIV‐negative/unknown gay/homosexual, bisexual or other cisgender men who have sex with men (GBM) and HIV‐negative/unknown other populations (POP), Brazil, 2019

	PLHIV (N = 347/1690, 20.5%)		GBM (N = 785/1690, 46.5%)		POP (N = 558/1690, 33.0%)	
	Rated completely/partially accurate 310 (90.1%)	aOR (95%CI)	Rated completely/partially accurate 487 (68.2%)	aOR (95%CI)	Rated completely/partially accurate 172 (34.5%)	aOR (95%CI)
Gender						
Cisgender men	244 (90.7)	Ref.	487 (68.2)	NA	28 (29.5)	Ref.
Cisgender women	56 (87.5)	0.80 (0.19 to 3.21)	0	NA	129 (33.8)	1.13 (0.70 to 1.84)
Transgender/ non‐binary	10 (90.9)	0.88 (0.19 to 6.34)	0	NA	15 (71.4)	**2.84 (1.07 to 7.72)** *
Sexual orientation						
Gay	227 (90.8)	1.72 (0.58 to 4.55)	409 (73.7)	**2.55 (1.81 to 3.58)** ****	8 (36.4)	0.50 (0.19 to 1.27)
Bisexual	20 (87.0)	Ref.	76 (48.7)	Ref.	24 (60.0)	Ref.
Heterosexual	60 (88.2)	3.78 (0.70 to 21.28)	0	NA	139 (32.0)	**0.50 (0.26 to 0.95)** *
Age						
≤35 years	121 (96.8)	**3.13 (1.65 to 6.22)** ****	305 (71.6)	**1.84 (1.37 to 2.48)** ****	59 (47.2)	**1.65 (1.09 to 2.47)** **
>35 years	189 (86.3)	Ref.	182 (63.2)	Ref.	113 (30.3)	Ref.
Ethno‐racial identity						
Black	39 (84.8)	**0.32 (0.16 to 0.68)** **	58 (73.4)	1.40 (0.89 to 2.25)	18 (43.9)	1.10 (0.59 to 2.01)
Other ^a^	266 (90.8)	Ref.	421 (67.1)	Ref.	152 (34.2)	Ref.
Monthly Family Income						
Middle/High	195 (92.9)	1.61 (0.85 to 3.05)	345 (70.4)	**1.47 (1.06 to 2.04)** **	117 (31.4)	0.78 (0.50 to 1.20)
Low^b^	115 (85.8)	Ref.	142 (63.4)	Ref.	55 (44.0)	Ref.
Education						
≤Secondary level	126 (88.7)	1.02 (0.54 to 1.97)	140 (67.6)	1.01 (0.72 to 1.43)	64 (38.1)	1.01 (0.67 to 1.53)
>Secondary level	183 (91.5)	Ref.	346 (68.8)	Ref.	108 (32.9)	Ref.
Residence in State Capital Metro area						
Yes	228 (89.4)	1.25 (0.66 to 2.31)	365 (70.1)	**1.42 (1.04 to 1.93)** *	123 (38.3)	1.42 (0.97 to 2.10)
No	82 (92.1)	Ref.	122 (63.2)	Ref.	49 (27.7)	Ref.
Steady partner						
Yes	118 (93.7)	**2.63 (1.39 to 5.27)** **	136 (66.3)	1.05 (0.77 to 1.44)	115 (35.2)	1.10 (0.75 to 1.63)
No	192 (88.1)	Ref.	351 (69.0)	Ref.	57 (33.3)	Ref.
Ever tested for HIV						
Yes	NA	NA	427 (69.3)	1.44 (0.94 to 2.21)	135 (38.5)	**1.73 (1.14 to 2.65)** **
No	NA	NA	60 (61.2)	Ref.	37 (25.2)	Ref.

NA, not applicable.

Bold indicates statistical significance (*p* < .05).

^a^ Other = White, Asian, Native or Pardo (Mixed‐Black) identities

^b^ low income is equivalent to R$1996.00 or USD468.00 per month.

**** *p* < .0001

* *p* < .01

* *p* < .05.

Our study adds to the body of evidence suggesting that perceived accuracy of U = U prevention benefits is low among populations most vulnerable to HIV, despite almost a decade since the first evidence of the effectiveness of TasP was published [25]. Our results confirm the gradient of greatest perceived accuracy of U = U among PLHIV compared to HIV‐uninfected/unknown [14, 24] and expands what is known to include cisgender women and heterosexual men with even lower perceived accuracy of all groups. Among the general population, ~11% did not know what “undetectable” means and, of those who rated the accuracy of U = U, only 17% rated it as completely accurate. On the other hand, among PLHIV, <1% did not know what “undetectable” means and 79% rated it as completely accurate. Though most of the prevention benefits from U = U will be realized from an accurate understanding of U = U among PLHIV and those most vulnerable to HIV, we echo the need for a broader understanding as suggested by others [13]. Indeed, recent findings highlight how implicit and explicit stigma of healthcare professionals towards PLHIV may be a key driver of the lack of U = U communication [8, 9]. HIV‐related stigma negatively impacts HIV prevention and treatment [8, 17], and, more generally, stigma has been proposed as fundamental cause of health inequality [20].

Compared to the largest and most recent study conducted among sexual minority GBM in the United States reporting that 54% and 39% of HIV‐negative and unknown GBM perceived U = U as completely or somewhat accurate [14], GBM assessed in our study showed greater perceived accuracy of U = U (68% rated it as completely or somewhat accurate). The above cited study has shown that perceived accuracy of U = U has increased with time [14], a finding that may suggest our results to have been influenced by the same pattern, as our study was conducted more than a year later (October 2019) than the US study [14]. Alternatively (or additionally), the greater perceived accuracy as observed in our study may result from local campaigns addressing the U = U slogan. Prior to this study, few campaigns addressing the U = U slogan have been promoted on YouTube channels (~100 000 views) and these campaigns have focused almost exclusively on PLHIV and GBM. GBM may have also been exposed to information on what it means to be undetectable as the Grindr app that we used for recruitment provides this information in a “health tab.” Furthermore, annually, the Department of Chronic Conditions and Sexually Transmitted Infections of the Ministry of Health has conducted campaigns launched on December 1st (World AIDS Day), which have gradually, since 2011, targeted GBM and focused on combination HIV prevention, including TasP [26]. These campaigns may have influenced our findings, especially the PLHIV and GBM groups.

Our model results showed that among PLHIV, Black identity was associated with significantly lower odds of perceiving U = U as completely accurate, possibly suggesting how multiple forms of stigma, come together to broadly influence health outcomes [27]. In a study conducted in Belo Horizonte, the sixth largest city in Brazil, Black persons had an almost twofold higher odds of experiencing discrimination than those of White identities, even after controlling for income, education, social status and health problems [28]. The confluence of two stigmatized identities, HIV‐infection and Black identity, may potentiate medical distrust and discrimination leading to an increased likelihood of information being withheld by medical providers. For GBM, we found that bisexual men were less likely to endorse U = U as completely accurate, possibly an unintended consequence of prevention/health promotion education campaigns that may be differentially accessed and/or received by bisexuals and other non‐gay identified men who have sex with men, as previously suggested [11]. Accordingly, there may be space for improvement in the messaging (from “reduces the risk of transmission” to “prevents HIV transmission” [14]) for U = U, for both gay‐ and non‐gay‐identified men who have sex with men, for other groups disproportionately affected by HIV and more broadly, for the general public. Community engagement could be beneficial in increasing awareness and acceptance of U = U among GBM of low income that may not be reached by online campaigns. For the general population, advertisements on mass media, such as TV and newspaper, could be an option. Lastly, consistent delivery of U = U education on the part of healthcare providers is of utmost importance, and clear and concise guidelines have been proposed [6].

This study has limitations. First, for this web‐based survey we did not use probabilistic sampling strategies but rather a convenience sample of those who have access to cell phones and use GSN apps or social media, resulting in a selective recruitment and hindering generalization of the findings. We believe, nevertheless, this type of selection bias might be small as recent data show that 85% of Brazilians have mobile phones and 79% have access to internet. Nonetheless, when comparing our sample of PLHIV with the characteristics of PLHIV in Brazil [29], our sample had less cisgender women (19.4% vs. 27.7%), more Black persons (13.7% vs. 10.5%), and were more educated (42% had more than secondary education vs. 18%). Our sample of POP, compared to the general population, was 80.6% cisgender women (vs. 52%), 11% were Black (vs. 9.4%) and 35% had more than secondary education (vs. 21%). For our main outcome, we only asked participants to rate the accuracy of one statement and, within response options, only acknowledged lack of understanding of the word undetectable. It may well be that the word “untransmittable” or the slogan altogether were misunderstood. Further probing the understanding of HIV viral load and how it relates to the sexual transmission of HIV or even adding a thorough explanation of the slogan may have yielded a different or more nuanced response. Though we actively searched for publications addressing the occurrence and effectiveness of U = U or TasP campaigns in Brazil, we found no such studies. Our review of published campaigns mentioned above strongly suggests that the populations most likely reached by these campaigns were PLHIV or GBM. Finally, all data were self‐reported by participants and may be subject to information bias, although individuals might be more honest through web‐based surveys, thereby reducing social desirability bias.

## 4 Conclusions

We note an urgent need for further educational strategies regarding TasP and U = U in Brazil. Our findings show that over half of HIV‐negative/unknown cisgender GBM and a staggering 82% of HIV‐negative/unknown cisgender women, heterosexual men and other populations perceived the slogan U = U inaccurately. Similar to prior studies conducted in high‐income settings, our results suggest higher knowledge among those living with HIV though it is important to highlight that 20% of our sample of PLHIV still perceived U = U as inaccurate. The finding that predictors of U = U perceived accuracy vary by group can help identify subgroups for whom further educational efforts are needed to enhance the individual and populational prevention benefits of antiretroviral treatment.

## Competing Interest

The authors declare no conflict of interest.

## Author Contributions

TST, LMSM, DRBB and PML conceived and implemented the survey. TST and PML analysed the data and generated the figure and tables. JC, VGV and BJ aided in the interpretation of results and its broader implications. TST and PML reviewed the literature and drafted the manuscript. All authors critically revised the manuscript for important intellectual content and approved the final version of the manuscript.

## Funding

Dr. Luz was supported by Programa Inova FIOCRUZ, Edital Geração do Conhecimento/VPPCB, and Programa PrInt, Coordenação de Aperfeiçoamento de Pessoal de Nível Superior (CAPES)/FIOCRUZ. Dr. Torres was supported by Conselho Nacional de Desenvolvimento Científico e Tecnológico (CNPq, #28/2018).
